# Supplementary material for: Modelling transcriptional silencing and its coupling to 3D genome organisation
Source: Soft Matter. 2025 Aug 6;21(35):6975–83. doi: 10.1039/d5sm00686d (PMC12371520; doi:10.1039/d5sm00686d)
Supplement: SM-021-D5SM00686D-s001 [file SM-021-D5SM00686D-s001.pdf]

Supplementary Information (SI) for Soft Matter.
This journal is © The Royal Society of Chemistry 2025

Soft Matter

Modelling transcriptional silencing and its coupling to 3D genome organisation – Electronic Supplementary Information –

M. Semeraro,[a†] G. Negro,[b*†] D. Marenduzzo,[b] and G. Forte[b]

The present document represents the *Electronic Supplementary Information* for the paper *Modelling transcriptional silencing and its coupling to 3D genome organisation*. Here we describe in depth the polymer model we adopt, report simulation and sampling details and show how simulation units map to physical ones. In addition, we also present supplementary figures which support and complement our discussion in the main paper.

1 Model

The polymer model we adopt is based on the *Diffusion Transcription Factors Model*[1–3]. Chromatin filaments are depicted as coarse-grained bead-and-spring polymers whose M beads are assumed to contain $1000-3000$ base pairs ($1-3\ kbp$), providing a bead diameter $\sigma \sim 20-30\ nm$[1]. The polymer is initially composed of two types of beads representing non-specific sites, i.e. low-affinity beads, and active Transcription Units (TUs), i.e. high-affinity beads, the latter interpreted as gene promoters or enhancers. We consider the same 1000 beads long polymer chain with 39 TUs placed randomly as in Ref. [2,3] and as depicted in Fig. S1(A) (the exact location of the TUs along the chain is reported in section 2). Transcription Factors (TFs) are modeled as N additional diffusing beads of diameter σ which can interact differently with the different beads of the polymer as detailed below. TFs are divided into N_a active and N_r repressive, so that $N = N_a + N_r$. Repressive TFs feature the capability of silencing TUs they are bound to with a given probability p_s, thus generating repressed TUs, which can revert back to active TUs after a fixed time interval τ_R. As schematically depicted in Fig. S1(B), both active and repressive TFs switch back and forth between an *ON* and *OFF* state at rates $\alpha_{on} = \alpha_{off}$ with a certain probability p_{switch}. As a consequence, on average there are $N_{a,on} = N_a \alpha_{on}/(\alpha_{on}+\alpha_{off})$ on-state and $N_{a,off} = N_a \alpha_{off}/(\alpha_{on}+\alpha_{off})$ off-state active TFs, with $N_a = N_{a,on} + N_{a,off}$. Similarly, on average there are $N_{r,on} = N_r \alpha_{on}/(\alpha_{on}+\alpha_{off})$ on-state and $N_{r,off} = N_a \alpha_{off}/(\alpha_{on}+\alpha_{off})$ off-state repressive TFs, with $N_r = N_{r,on} + N_{r,off}$. When in the *OFF* state, active and repressive TFs experience a purely steric interaction with the whole chromatin filament, while active *ON* TFs experience strong attractive interactions with active TUs (hence the high-affinity denomination) and weak ones with all other chromatin beads, i.e. non-specific sites and repressed TUs (hence the low-affinity denomination). As for interactions between repressive *ON* TFs and chromatin beads, we consider three different feedback scenarios, each mimicking a different silencing mechanism:

- *positive feedback*, Fig. S1C. Repressive *ON* TFs experience a strong attraction to active and repressed TUs, and a weak attraction to non-specific sites. This represents a *color and stick* scenario as here repressive TFs bind to active TUs, repress them and keep being highly attracted to repressed TUs, which are less likely to become again accessible and attractive to active TFs.
- *negative feedback*, Fig. S1D. Repressive *ON* TFs sterically interact with most chromatin beads, except for active TUs, which are considered to be strongly attractive. This represents a *color and flee* scenario as here repressive TFs usually detach from the chain after silencing active TUs.
- *neutral feedback*, Fig. S1E. Repressive ON TFs are weakly attracted to the whole chromatin filament, independently of the bead type. This represents a *color and linger* scenario as repressive TFs keep hanging around the chain even after silencing active TUs.

[a] *Dipartimento Interateneo di Fisica, Università degli Studi di Bari and INFN, Sezione di Bari, via Amendola 173, Bari, I-70126, Italy*
[b] *SUPA School of Physics and Astronomy, University of Edinburgh, Peter Guthrie Tait Road, Edinburgh EH9 3FD, UK*
† These authors contributed equally to this work.
∗ giuseppe.negro@ba.infn.it

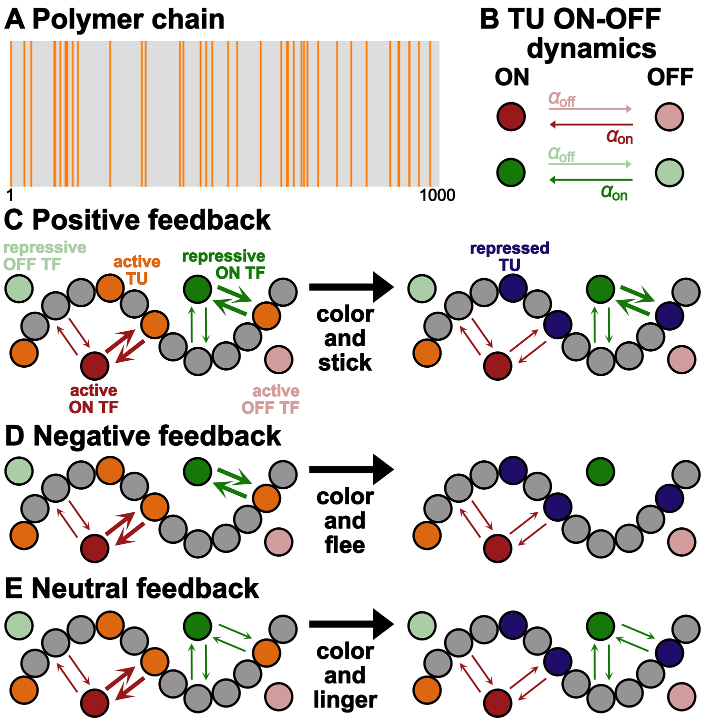

Fig. S1 (A) Initial polymer bead sequence. Orange and gray lines denote the location of active TUs and unmarked chromatin sites respectively. Bead ID number ranges between 1 and 1000. (B) Sketch of the *ON-OFF* dynamics of active (red) and repressive (green) TFs occurring with switching rates $\alpha_{on} = \alpha_{off}$. TFs in the *OFF* state only interact sterically with the chromatin filament. Interactions between chromatin and *ON* TFs depend on the silencing feedback mechanism which is assumed in the model. (C) to (E) Schematic representation of the three silencing models (positive, negative and neutral feedback). The chromatin filament is represented as polymer formed by a sequence of connected beads. Active and repressed TUs are depicted as orange and blue beads respectively, while unmarked chromatin is represented by gray beads. *ON* and *OFF* active (repressive) TFs are instead depicted as dark and light red (green) beads respectively. Thicker and thinner arrows denote strong and weak attractive interactions. In the *positive feedback* model (panel (C)) repressive *ON* TFs are strongly attracted to both active and repressed TUs, and weakly to unmarked chromatin beads. In the *negative feedback* model (panel (D)) repressive *ON* TFs are strongly attracted only to active TUs, while in *neutral feedback* (panel (E)) they are weakly attracted to the whole chromatin filament. In all three feedback mechanisms active TFs are strongly attracted to active TUs and experience a weak attraction to repressed TUs and unmarked chromatin.

Let us now detail how bead dynamics and interactions are implemented. We introduce a discrete index i running from 1 to $M+N$ for polymer beads, and active and repressive TFs. The symbol $\mathbf{r}_i = (r_{ix}, r_{iy}, r_{iz})$ will thus denote the position of the i-th generic bead in the three-dimensional space, while $r_{ij} = \sqrt{\sum_{\beta=x,y,z}(r_{i\beta} - r_{j\beta})^2}$ the Euclidean distance between the i-th and the j-th beads. Concerning polymer beads, the generic i-th and j-th beads interact sterically via the truncated Weeks-Chandler-Anderson (WCA) potential[4]

$$U^{ij}_{WCA} = \begin{cases} 4k_BT\left[\left(\frac{\sigma}{r_{ij}}\right)^{12} - \left(\frac{\sigma}{r_{ij}}\right)^{6}\right] & \text{if } r_{ij} < 2^{1/6}\sigma \\ 0 & \text{otherwise} \end{cases}, \tag{1}$$

where k_B is the Boltzmann constant and T is the environment temperature. In order to enforce chain connectivity, any two consecutive polymer beads additionally interact via a finitely extensible non-linear elastic (FENE) potential

$$U^{ij}_{FENE} = -\frac{k_f R_0^2}{2}\ln\left[1 - \left(\frac{r_{ij}}{R_0}\right)^2\right], \tag{2}$$

where k_f is the spring constant and R_0 is the maximum separation between the beads. Finally, the stiffness of the chromatin filament is taken into account by making each three consecutive beads along the polymer interact via the Kratky-Porod potential

$$U^{ij}_{KP} = \frac{k_BTl_P}{\sigma}\left[1 - \frac{\vec{s}_i \cdot \vec{s}_j}{|\vec{s}_i||\vec{s}_j|}\right] = k_{kp}\left[1 - \cos(\theta)\right] ,$$

where i and $j = i+1$ are neighboring beads, $\vec{s}_i$ is the tangent vector connecting the i-th and the $i+1$-th beads, θ is the angle formed by such tangents and and l_P is the persistence length of the chain.

A polymer bead with index i and an active/repressive TF with index j interact via the following WCA potential

$$U_{WCA}^{ab} = \begin{cases} 4\varepsilon_{ij}\left[\left(\frac{\sigma}{r_{ij}}\right)^{12} - \left(\frac{\sigma}{r_{ij}}\right)^{6}\right] & \text{if } r_{ab} < r_c \\ 0 & \text{otherwise} \end{cases} .$$

The interaction cut-off is set to $r_c = 2^{1/6}\sigma$ for steric interactions and to $r_c = 1.8\sigma$ for weak and strong attractive interactions. In addition, we set $\varepsilon_{ij} = k_BT$ for steric interactions and $\varepsilon_{ij} = 3k_BT, 8k_BT$ for weak and strong attractive ones, respectively.

Finally, the global time evolution of the system is described by the solution of the following system of $3(N+M)$ Langevin equations

$$m_i \frac{d^2 r_{i,\alpha}}{dt^2} = -\nabla U_i - \gamma_i \frac{dr_{i,\alpha}}{dt} + \sqrt{2k_BT\gamma_i}\ \eta_{i,\alpha}(t) \ , \tag{3}$$

where i is the bead index running from 1 to $N+M$, $\alpha = x,y,z$ is the dimensional index, m_i and γ_i are the mass and friction coefficient associated to the i-th bead, U_i is the total potential experienced by the i-th bead and $\eta_{i,\alpha}(t)$ are a set of independent zero-mean delta-correlated stochastic white noises, i.e.

$$\langle \eta_{i,\alpha}(t) \rangle = 0 \ , \qquad \langle \eta_{i,\alpha}(t)\eta_{j,\beta}(t') \rangle = \delta_{ij}\delta_{\alpha\beta}\delta(t-t') \ ,$$

where δ_{ij} and $\delta_{\alpha\beta}$ are two Kronecker deltas over beads and dimensions, respectively, and $\delta(t-t')$ is a Dirac delta. For the sake of simplicity we set the mass $m_i \equiv m$ and friction coefficient $\gamma_i \equiv \gamma$, equal for all beads.

2 Numerical Methods

For the sake of comparison, as in Ref.[2,3] we consider a $M = 1000$ beads long polymer chain with 39 TUs placed randomly. Figure S1A offers a graphical depiction of the chromatin filament. The number of TUs is chosen in such a way that the ratio $n_{TU}/M \sim 0.04$ is of the same order as most human chromosomes[3]. More in detail, along the $M = 1000$ beads of the chain, TUs have chain bead indices 2, 33, 49, 103, 105, 117, 129, 133, 146, 158, 233, 307, 316, 394, 404, 444, 457, 508, 529, 584, 632, 645, 648, 661, 692, 679, 685, 693, 718, 762, 795, 831, 886, 905, 907, 930, 931, 953 and 979. Following this order, we refer to the TU index as varying from 1 to 39. In addition, we consider $N_a = N_r = 40$ TFs, such that $N_a < n_{TU}$ as observed in mammalian cells[1,5].

Coarse grained Brownian molecular dynamics (MD) simulations are performed using the LAMMPS (Large-scale Atomic/Molecular Massively Parallel Simulator) software package[6,7] (release 3Mar2020). We use the Velocity-Verlet algorithm with fix nve and the addition Langevin thermostat, i.e. the fix langevin, which models the stochastic thermal fluctuations and viscosity of an implicit solvent. For the sake of simplicity, hydrodynamic interactions are neglected and mass m, diameter σ, temperature T, and Boltzmann constant k_B are all set to unity. The typical timescales are the Lennard-Jones time $\tau_{LJ} = \sigma\sqrt{m/\varepsilon}$, with $\varepsilon = k_BT$ the energy unit, and the brownian time $\tau_B = \sigma^2/D$, with $D = k_BT/\gamma$ the diffusion coefficient of a single bead. Setting $\gamma = 1$, we have $\tau_B = \tau_{LJ} = 1$. Moreover, we fix $k_f = 30k_BT/\sigma^2$, $R_0 = 1.6\ \sigma$ and $l_P = 3.0\ \sigma$ as in[2,3].

The system of equations Equation 3 is integrated with timestep $\Delta t = 0.01\ \tau_B$. The simulation domain is a periodic cubic box of side $L = 100\sigma$ chosen so that the filament volume fraction is $\sim 4.2 \cdot 10^{-3}$ and the ones of active and repressive TFs are $\sim 1.7 \cdot 10^{-4}$, i.e. the system is dilute. Protein switching is implemented by stochastically changing each TF state every $n_s = 10^2\ \tau_B$ as prescribed by the switching off and on rates $\alpha_{off} = \alpha_{on} = 10^{-3}$. As a consequence, $N_{a,on} = N_{a,off} = N_{r,on} = N_{r,off} = 20$. Similarly to TFs, repressed TUs can switch back to an active state every $\tau_R = 10^2\ \tau_B$ with rate $\alpha_R = 10^{-3}$.

Simulations begin with the monomers of the chromatin filament initialised with a random walk, and both active and repressive TFs placed randomly within the simulation domain. Bead-bead overlaps are relaxed by first evolving the system for a few thousand timesteps with a soft repulsive potential between beads. The system is then thermalized for $10^5\ \tau_B$ with only repulsive WCA potential interactions between all beads. Finally the system is evolved for $8 \cdot 10^5\ \tau_B$ including weak and attractive interactions as well as transcriptional feedbacks, and the observables of interest are monitored. A TU is considered transcriptionally active if the distance between its centre and the centre of almost one active TF is less than 2.23 σ as in[3] (we checked that slightly varying the transcriptional threshold does not alter significantly the output transcriptional profiles). For each TU, transcriptional activity is thus evaluated as the fraction of timesteps it is in close proximity to almost one active TF. Averages are evaluated over 100 independent runs for each case. Clusters are identified using the python-implemented DBSCAN algorithm[8] with distance threshold equal to the threshold for transcriptional activity. Correlation maps and networks between TUs is obtained by evaluating the transcriptional activity Pearson correlation coefficient between each couple of TUs. For completeness, we recall that, given the two sets of data $X = \{x_i\}_{i=1,\dots,n}$ and $Y = \{y_i\}_{i=1,\dots,n}$, the Pearson correlation coefficient is defined as $cov(X,Y)/(\sigma_X\sigma_Y) \in [-1,1]$, where $cov(X,Y) = \sum_{i=1}^{n}(x_i - \bar{X})(y_i - \bar{Y})/n$ is the covariance between X and Y, with $\bar{X}, \bar{Y}$ and their respective average values and variances.

All the scripts and the LAMMPS fix needed to produce the results presented in the main text, are publicly available at `10.5281/zenodo.15800260`.

3 Mapping simulation units to physical units

Simulation units can be readily converted into physical units. Using the realistic values of bead diameter $\sigma = 20 - 30$ nm, Boltzmann constant $k_B \sim 1.38 \cdot 10^{-23}$ $m^2 kg/(s^2 K)$ room temperature $T = 300$ K and nucleoplasm viscosity $\eta_{sol} = 10 - 100$ cP, we find $\varepsilon = k_B T \simeq 1.38 \cdot 10^{-21}$ J and $\tau_{LJ} = \tau_B = \sigma^2/D = 3\pi\eta_{sol}\sigma^3/\varepsilon \simeq 5 \cdot 10^{-3} - 2 \cdot 10^{-1}$ s.

4 Supplementary Figures

In this section we include supplementary figures which support and complement our discussion in the main text.

In Figure S2 we report the trend of the average size of clusters formed by active TUs over time for the the positive, negative and neutral feedback models. Each curve is obtained averaging over 100 independent runs each lasting $8 \cdot 10^5$ $5\tau_B$.

In Figure S3 we report transcriptional activity profiles for positive, negative and neutral transcriptional feedback schemes. We remark that a TU bead is considered transcriptionally active whilst within $2.23\sigma \sim 4.5 - 6.7$ nm of an active TF, and also that the transcriptional activity for each TU $\langle a \rangle_{TU}$ is obtained averaging over 100 independent runs each lasting $8 \cdot 10^5 \tau_B$. For comparison, in all panels we consider also the case in which the corresponding transcriptional feedback is turned off, i.e. $p_s = 0$.

In Figure S4 we report a selection of kymographs relative to representatives runs for positive, negative and neutral transcriptional feedback (columns) and the different values of $p_s = 10^{-3}, 10^{-2}, 10^{-1}$ and $9 \cdot 10^{-4}$ (rows). In each panel, from bottom to top, each line is associated to subsequent TUs along the polymer chain. Black, yellow and red pixels denote a transcriptionally passive, active but not transcribing and active and transcribing state, respectively.

In Figure S5 we report the Pearson correlation matrix for the activity of all TUs in the chromatin filament for positive, negative and neutral transcriptional feedback (rows) and different values of p_s (columns). In each matrix, each TU is associated to a row and column, with the latter in turn ordered according to the TU index from low to high. Each ij pixel is coloured according to Pearson correlation value between the i-th and j-th TU pair according to the bars on the right. These plots are used to obtain the correlation networks in Fig.4 of the main text.

Feedback	Edge	$p_s = 10^{-4}$	$p_s = 10^{-3}$	$p_s = 10^{-2}$	$p_s = 10^{-1}$	$p_s = 9 \cdot 10^{-1}$
Positive	positive	140	82	158	246	224
	negative	88	78	92	28	10
Negative	positive	118	58	30	84	218
	negative	42	22	16	24	94
Neutral	positive	194	144	60	76	124
	negative	82	114	74	84	34

Table 1 Number of edges relative to positive and negative correlations above a threshold of 0.25, corresponding to a p-value ~ 2 10^{-2} for positive, for negative and neutral transcriptional feedback and the different values of p_s considered in Fig.4 of the main text.

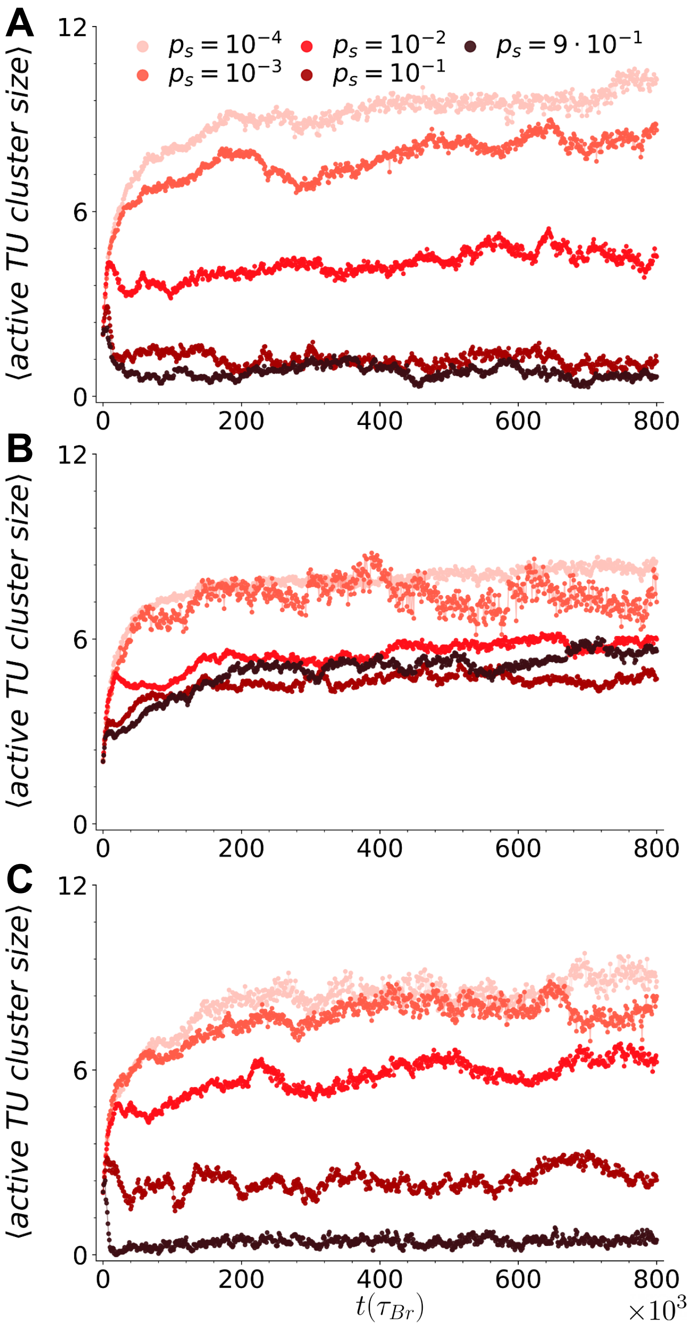

Fig. S2 (A) to (C): Average size of clusters formed by active TUs over time for the the positive, negative and neutral feedback models, respectively. Each curve is obtained averaging over 100 independent runs each lasting $8 \cdot 10^5$ $5\tau_B$. The darker the curve, the higher the silencing probability p_s it represents.

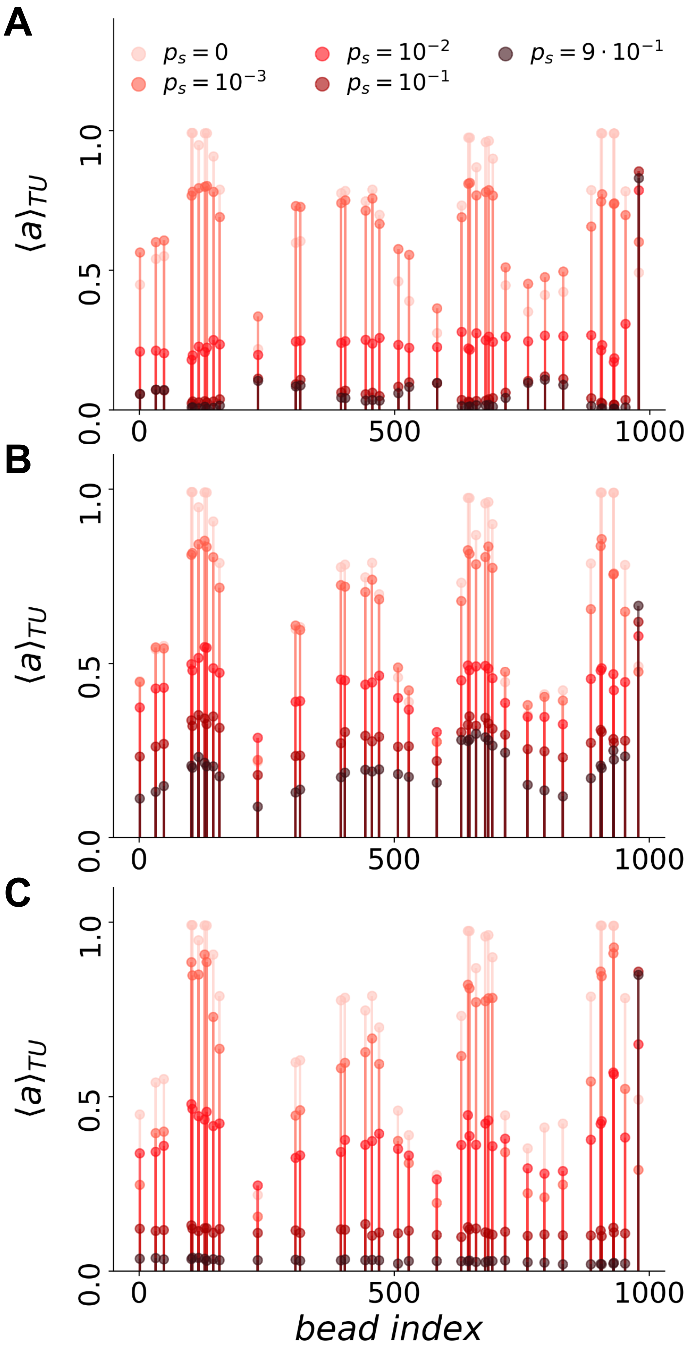

Fig. S3 (A) to (C): Transcriptional activity profile for positive, negative and neutral transcriptional feedback, respectively. Each $\langle a \rangle_{TU}$ value is obtained averaging over 100 independent runs each lasting $8 \cdot 10^5$ $5\tau_B$. A TU bead is considered to be active whilst within $2.23\sigma \sim 4.5 - 6.7$ nm of an active TF.

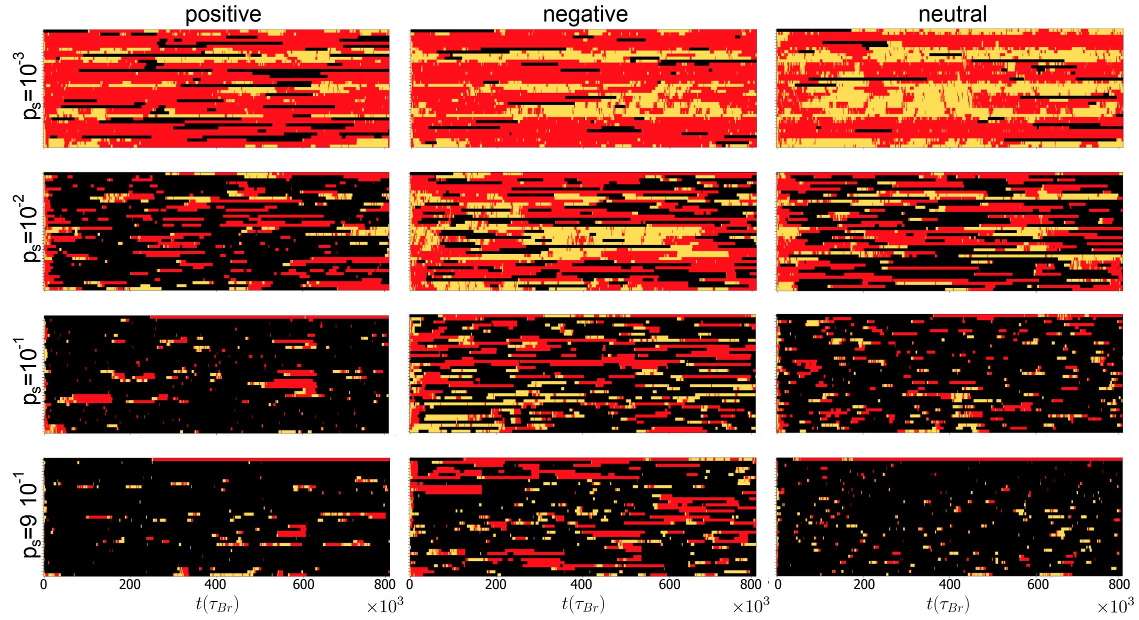

Fig. S4 Kymograph of representatives runs for positive, negative and neutral transcriptional feedback (columns) and different values of p_s (rows). From bottom to top, each line is a associated to subsequent TUs along the polymer chain. Black, yellow and red pixels denote a transcriptionally passive, active but not transcribing and active and transcribing state, respectively.

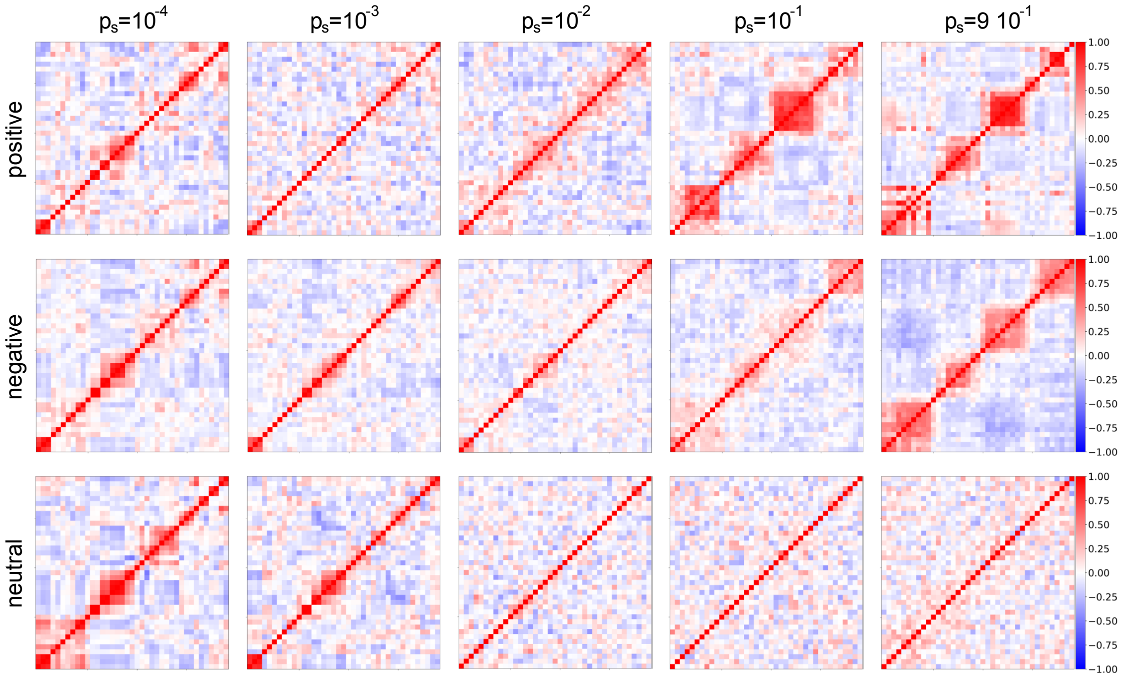

Fig. S5 Pearson correlation matrix for the activity of all TUs in the chromatin filament for positive, negative and neutral transcriptional feedback (rows) and different values of p_s (columns). In each matrix, each TU is associated to a row and column, with the latter in turn ordered according to the TU index from low to high. Each ij pixel is coloured according to Pearson correlation value between the i-th and j-th TU pair according to the bars on the right.

Notes and references

1 C. A. Brackley, J. Johnson, S. Kelly, P. R. Cook and D. Marenduzzo, *NAR*, 2016, **44**, 3503–3512.

2 C. Brackley, N. Gilbert, D. Michieletto, A. Papantonis, M. Pereira, P. Cook and D. Marenduzzo, *Nat. Comm.*, 2021, **12**, 5756.

3 M. Semeraro, G. Negro, A. Suma, G. Gonnella and D. Marenduzzo, *Physica A*, 2023, **625**, 129013.

4 J. D. Weeks, D. Chandler and H. C. Andersen, *J. Chem. Phys.*, 1971, **54**, 5237–5247.

5 R. C. Brewster, F. M. Weinert, H. G. Garcia, D. Song, M. Rydenfelt and R. Phillips, *Cell*, 2014, **156**, 1312–1323.

6 S. Plimpton, *J. Comp. Phys.*, 1995, **117**, 1–19.

7 *LAMMPS Molecular Dynamics Simulator*, `https://www.lammps.org/#gsc.tab=0`.

8 *Python DBSCAN algorithm*, `earn.cluster.DBSCAN.html`.
